# Profiling Small RNA From Brain Extracellular Vesicles in Individuals With Depression

**DOI:** 10.1093/ijnp/pyae013

**Published:** 2024-03-08

**Authors:** Pascal Ibrahim, Ryan Denniston, Haruka Mitsuhashi, Jennie Yang, Laura M Fiori, Dariusz Żurawek, Naguib Mechawar, Corina Nagy, Gustavo Turecki

**Affiliations:** Integrated Program in Neuroscience, McGill University, Montreal, Quebec, Canada; McGill Group for Suicide Studies, Douglas Mental Health University Institute, Verdun, Quebec, Canada; McGill Group for Suicide Studies, Douglas Mental Health University Institute, Verdun, Quebec, Canada; Integrated Program in Neuroscience, McGill University, Montreal, Quebec, Canada; McGill Group for Suicide Studies, Douglas Mental Health University Institute, Verdun, Quebec, Canada; McGill Group for Suicide Studies, Douglas Mental Health University Institute, Verdun, Quebec, Canada; McGill Group for Suicide Studies, Douglas Mental Health University Institute, Verdun, Quebec, Canada; McGill Group for Suicide Studies, Douglas Mental Health University Institute, Verdun, Quebec, Canada; Integrated Program in Neuroscience, McGill University, Montreal, Quebec, Canada; McGill Group for Suicide Studies, Douglas Mental Health University Institute, Verdun, Quebec, Canada; Department of Psychiatry, McGill University, Montreal, Quebec, Canada; Integrated Program in Neuroscience, McGill University, Montreal, Quebec, Canada; McGill Group for Suicide Studies, Douglas Mental Health University Institute, Verdun, Quebec, Canada; Department of Psychiatry, McGill University, Montreal, Quebec, Canada; Integrated Program in Neuroscience, McGill University, Montreal, Quebec, Canada; McGill Group for Suicide Studies, Douglas Mental Health University Institute, Verdun, Quebec, Canada; Department of Psychiatry, McGill University, Montreal, Quebec, Canada

**Keywords:** Extracellular vesicles, miRNA, depression, size exclusion chromatography, ultracentrifugation

## Abstract

**Background:**

Major depressive disorder (MDD) is a leading cause of disability with significant mortality risk. Despite progress in our understanding of the etiology of MDD, the underlying molecular changes in the brain remain poorly understood. Extracellular vesicles (EVs) are lipid-bound particles that can reflect the molecular signatures of the tissue of origin. We aimed to optimize a streamlined EV isolation protocol from postmortem brain tissue and determine whether EV RNA cargo, particularly microRNAs (miRNAs), have an MDD-specific profile.

**Methods:**

EVs were isolated from postmortem human brain tissue. Quality was assessed using western blots, transmission electron microscopy, and microfluidic resistive pulse sensing. EV RNA was extracted and sequenced on Illumina platforms. Functional follow-up was performed *in silico*.

**Results:**

Quality assessment showed an enrichment of EV markers, as well as a size distribution of 30 to 200 nm in diameter, and no contamination with cellular debris. Small RNA profiling indicated the presence of several RNA biotypes, with miRNAs and transfer RNAs being the most prominent. Exploring miRNA levels between groups revealed decreased expression of miR-92a-3p and miR-129-5p, which was validated by qPCR and was specific to EVs and not seen in bulk tissue. Finally, *in silico* functional analyses indicate potential roles for these 2 miRNAs in neurotransmission and synaptic plasticity.

**Conclusion:**

We provide a streamlined isolation protocol that yields EVs of high quality that are suitable for molecular follow-up. Our findings warrant future investigations into brain EV miRNA dysregulation in MDD.

**Significance Statement**

Understanding the molecular mechanisms behind major depressive disorder (MDD), a leading global cause of disability, is the crucial first step toward the development of urgently needed new treatments. Here, we optimized a streamlined way of investigating extracellular vesicles (EVs) from postmortem human brain. We identified that microRNAs (miRNAs) are the most abundant RNA species in brain-derived EVs. We also found that miR-92a-3p in females and miR-129-5p in males were less abundant in EVs from MDD subjects and are both involved in neurotransmission and synaptic plasticity. This study serves as a starting point toward understanding the possible role of EVs in the pathophysiology of MDD, which could potentially help identify novel therapeutic targets and biomarkers for this debilitating disease.

## INTRODUCTION

Major depressive disorder (MDD) is a leading cause of disability, affecting approximately 280 million people worldwide, with suicide as the most severe outcome (Institute of Health Metrics and Evaluation, 2021; World Health Organization., 2021). Although effective treatments exist for MDD, the response rates are not ideal, the latency to pharmacological action is long, and there could be adverse side effects (Otte et al., 2016). Novel treatments are urgently needed to face this global burden. To date, our understanding of the psychopathology of MDD remains limited, and more effort must be focused on deciphering the core brain molecular and functional alterations underlying the disease to develop more effective treatments.

Extracellular vesicles (EVs) are membrane-bound compartments produced and released by cells into various bodily fluids. There are different types of EVs, which are differentiated by their size and biogenesis pathways, including exosomes, microvesicles, and apoptotic bodies (Hessvik and Llorente, 2018). For this study, we refer to EVs as small vesicles ranging from 30 to 200 nm in diameter, regardless of biogenesis pathway. EVs carry cargo from their cell of origin and thus partially reflect the identity and state of that cell (Colombo et al., 2014). There is also evidence of EV uptake by recipient cells, potentiating cargo release into the cells followed by a change in function, which suggests a role of EVs in intercellular communication (Mulcahy et al., 2014).

Interestingly, EVs have been found to mediate intercellular communication between cells in the CNS (Bakhti et al., 2011; Bahrini et al., 2015; Gabrielli et al., 2015; Pascua-Maestro et al., 2018), which leads to the possibility that they play a role in the physiology and pathophysiology of the CNS. Investigating brain EVs can be a means for identifying novel molecular alterations in the context of brain disease. Accumulating evidence is emerging around the role of EVs in Alzheimer’s (Rajendran et al., 2006; Wang et al., 2012), Parkinson's (Chang et al., 2013; Stuendl et al., 2016), amyotrophic lateral sclerosis (Basso et al., 2013; Iguchi et al., 2016), and psychiatric disorders, including MDD (Wei et al., 2020; Goetzl et al., 2021; Saeedi et al., 2021; Wang et al., 2021), schizophrenia (Du et al., 2019), bipolar disorder (Banigan et al., 2013), and autism spectrum disorder (ASD) (Tsilioni and Theoharides, 2018).

To date, there are no studies to our knowledge examining EVs in the human brain of individuals with depression. In this study, we aimed to investigate brain EV differences associated with MDD, focusing on the EV microRNA (miRNA) cargo, which is the most studied RNA biotype in EVs. We chose the anterior cingulate cortex (ACC) as our region of interest, as it is highly implicated in MDD (Fiori et al., 2021). First, we demonstrated successful isolation of EVs from postmortem human brain tissue using size exclusion chromatography (SEC). We then profiled the EV miRNA cargo and compare these profiles between depressed suicide cases and psychiatrically healthy controls. We found certain miRNAs showed a tendency toward lower expression in depression. To determine the possible functional consequences of alterations to these miRNAs, we performed an exploratory *in silico* analysis, which pointed to effects in neurotransmission and synaptic plasticity. This is the first study, to our knowledge, to profile miRNA cargo in brain-derived EVs in the context of depression. This study can help identify novel therapeutic targets as well as potential biomarkers that could aid in the diagnosis of MDD and the subsequent prevention of suicide.

## MATERIALS AND METHODS

### Human Brain Samples

Frozen postmortem ACC samples (Brodmann area 24) were obtained from the Douglas-Bell Canada Brain Bank. Tissue dissections were performed as previously described (Lutz et al., 2017). This study included 80 subjects, 40 [20 male (M)/20 female (F)] of whom were psychiatrically healthy controls who died suddenly without a prolonged agonal state (CTRL group). The other 40 subjects (20 M/20 F) died by suicide in the context of a major depressive episode (MDD group). Table 1 provides more information on the subject groups. Psychological autopsies were performed by trained clinicians using structured diagnostic interviews, as previously described (Dumais et al., 2005), with the informants best acquainted with the deceased, and DSM IV criteria were used for diagnostic purposes. The research ethics board of the Douglas Mental Health University Institute provided ethical approval for this study, and the families of the deceased provided written informed consent.

**Table 1.** Cohort description

	Males		Females	
	CTRL	MDD	CTRL	MDD
Number	20	20	20	20
Age (y)	42 ± 4	52 ± 3	68 ± 3	55 ± 3^*a*^
PMI (h)	32 ± 5	46 ± 4^*a*^	56 ± 6	49 ± 4
Tissue pH	6.55 ± 0.05	6.58 ± 0.06	6.29 ± 0.07	6.50 ± 0.08
BMI	30.6 ± 2.8	24.7 ± 1.4	24.3 ± 1.6	24.2 ± 1.5
Race	Caucasian	Caucasian	Caucasian	Caucasian (18), Hispanic (1), N/D (1)
Cause of death	Natural (9)/accident (11)	Suicide	Natural (15)/accident (5)	Suicide
Substance use disorder (%)	0 (0%)	12 (60%)	1 (5%)	2 (10%)
Psychiatric medication (%)	None	Antidepressants 6 (30%), Benzodiazepines 7 (35%), Antipsychotics 1 (5%), Other 1 (5%)	Antidepressants 1 (5%), Benzodiazepines 2 (10%), Antipsychotics 1 (5%), Other 1(5%)	Antidepressants 12 (60%), Benzodiazepines 6 (30%), Antipsychotics 2 (10%), Other 5 (25%)

Abbreviations: BMI, body mass index; MDD, major depressive disorder; N/D, not determined; PMI, postmortem interval. Values represent Mean ± SEM. ^*a*^*P* < .05 for Mann-Whitney U test or Student *t* test.

### EV Isolation

#### Tissue Homogenization

**Figure 1.**
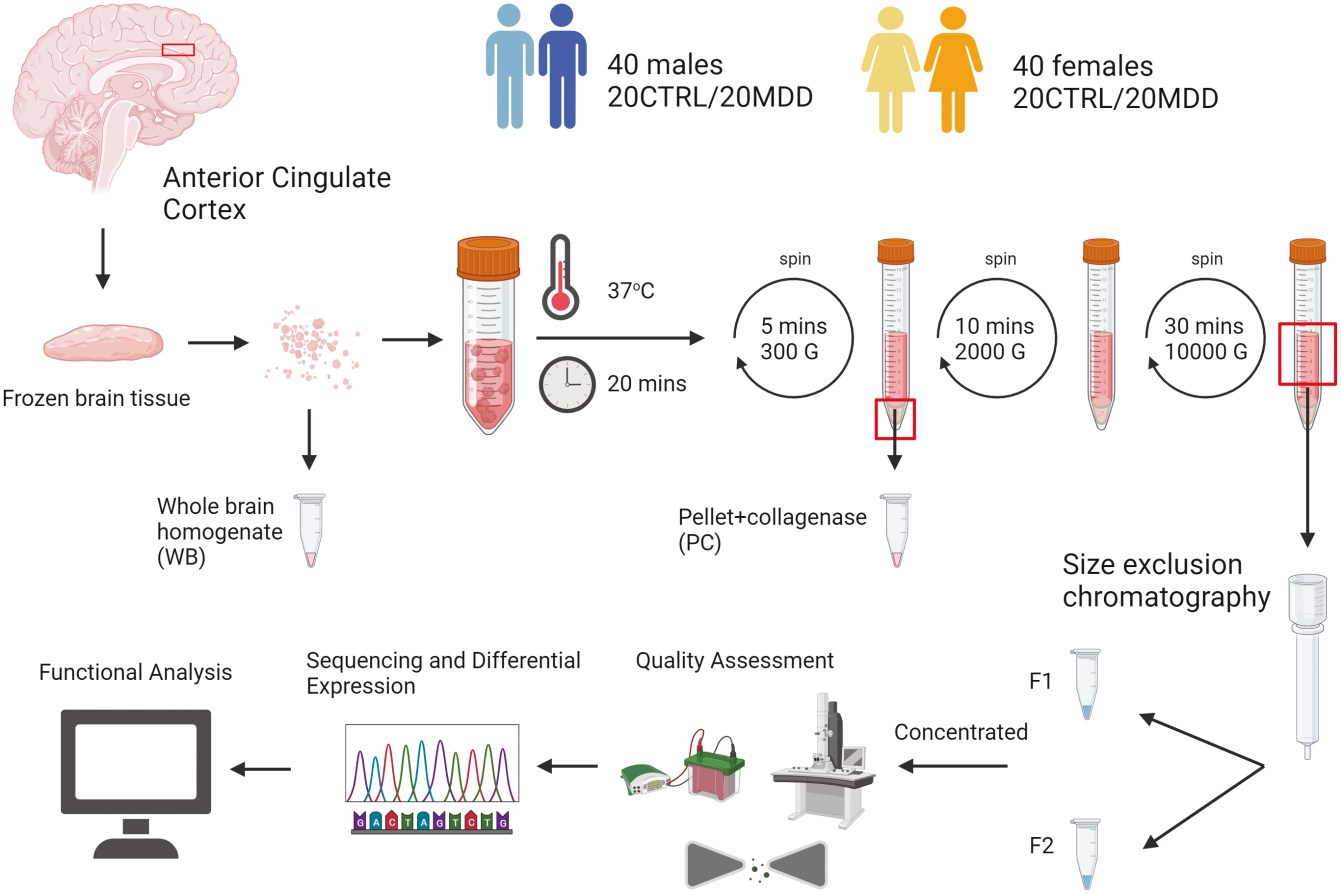
Schematic diagram representing study workflow. CTRL, control; F1, fraction 1; F2, fraction 2; MDD, major depressive disorder. Figure was created using BioRender.

For each subject, 200 to 400 mg of brain tissue were cut into very fine pieces while still partially frozen. The tissue was then incubated in Hibernate E medium (ThermoFisher Scientific, Waltham, Masschusetts, USA) (800 µL/100 mg of tissue) in the presence of 75 U/mL of collagenase type III (Worthington Biochemical, Freehold, New Jersey, USA) at 37°C for 20 minutes with gentle shaking. The reaction was then halted using protease (PI) and phosphatase (PS) inhibitors (1×) (Millipore-Sigma, Burlington, Massachusetts, USA), and the sample was centrifuged at 300 *g* for 5 minutes. The pellet was homogenized in phosphate buffered saline (PBS) with 1X PI/PS, while the supernatant was collected and centrifuged at 2000 g for 10 minutes. The supernatant was collected again and centrifuged at 10 000 *g* for 30 minutes (Vella et al., 2017). We isolated EVs using 2 methods: ultracentrifugation (UC) and sucrose gradient for test samples, and SEC for our cohort. Ten milligrams of tissue was also homogenized in PBS with 1X PI/PS and stored at −80°C. Figure 1 shows a schematic of the workflow.

#### Ultracentrifugation and Sucrose Gradient

The supernatant of test samples was overlaid on a sucrose gradient (0.6 M, 1.3 M, 2,5 M). The gradient was prepared starting with 1 mL of 2.5 M sucrose, which was overlaid with 1.2 mL of 1.3 M sucrose followed by 1.2 mL of 0.6 M sucrose. The exact volume of the supernatant was recorded (X mL) and overlaid onto the gradient, which was then spun (Beckman Optima XPM-100; swinging bucket rotor SW41 Ti) for 3 hours at 180 000 *g* (UC) at 4°C. After UC, the top layer of the gradient was discarded (X mL—1.2 mL = volume removed). The next 1.2 mL was designated as fraction 1 (UC-F1), which is expected to contain large vesicles between 200 and 1000 nm. Fraction 2 (UC-F2; 1.2 mL) and 3 (UC-F3; 1.2 mL) were subsequently collected and were expected to contain vesicles between 50 and 200 nm and 20 and 30 nm, respectively. Each fraction was diluted with PBS and spun at 100 000 *g* for 1 hour at 4°C. The supernatant was then discarded, and the pellets were collected in PBS with 1X PI/PS.

#### Size Exclusion Chromatography

The supernatant of cohort samples was overlaid on a size exclusion column (Izon Science, New Zealand) for EV separation according to the manufacturer’s protocol. qEV original columns of the 70-nm series, which are optimized for isolation of particles 70 to 1000 nm in size, were used. Fractions 7, 8, and 9 were collected and labeled as F1 (EV fraction), and fractions 10, 11, and 12 were collected and labeled as F2 (protein fraction). An aliquot of F1 was taken for measuring the size distribution of vesicles. F1 and F2 were then concentrated using Amicon centrifugal filters (Millipore-Sigma, USA).

### EV Size Distribution

Microfluidic resistive pulse sensing was used to measure the size distribution of the EVs in F1. A subsample of 10 males (5 CTRL/5 MDD) and 10 females (5 CTRL/5 MDD) was used. Measurements were taken using the nCS1 instrument (Spectradyne, Signal Hill, California, USA) and acquired using the Spectradyne Acquisition Software version 2.5.0.261. A total of 5 μL of each sample was run once with a C-400 cartridge, which detects particles 65 to 400 nm in diameter (anything smaller than 65 nm is treated as noise). The average concentration per particle size was then calculated and plotted.

### Western Blot

Proteins were extracted from different fractions of SEC as well as UC, whole brain homogenate (WB), and homogenized pellet with collagenase type III (PC). The samples were lysed [150 mM NaCl, 50 mM HEPES (pH 7), 50 mM Ethylenediamine tetraacetic acid (EDTA), and 0.1 % NP-40] and sonicated. The samples were clarified (13 000 rpm for 5 minutes at 4°C) and quantified using a Bicinchoninic acid (BCA) assay (ThermoFisher Scientific, USA). Equivalent amounts of proteins were run on a 4% to 20% Mini-PROTEAN TGX Stain-Free Gels (Bio-Rad, Hercules, California, USA). The gels were then activated with UV light to visualize total protein loading (Stain-Free method) using a Bio-Rad ChemiDoc MP imaging system before proteins were transferred onto nitrocellulose membranes (Bio-Rad, USA). The membranes were blocked with 5% nonfat dry milk (NFDM) in PBS with 0.05% Tween 20 (PBS-T) at room temperature for 1 hour and then incubated with primary antibodies Calnexin (Abcam, UK, Cat#ab22595), BiP (BD Biosciences, Franklin Lakes, New Jersey, USA, Cat#610978), VDAC (Cell Signaling Technology, Danvers, Massachusetts, USA, Cat#4661T), CD9 (System Biosciences, Palo Alto, California, USA, Cat#EXOAB-KIT-1), and TSG101 (Sigma, USA, Cat#HPA006161) diluted 1:500 in 1% NFDM in PBS-T overnight at 4°C. They were then incubated with biotin-conjugated secondary antibody (Vector Laboratories, Newark, California, USA) diluted 1:500 in 1 % NFDM in PBS-T for 1 hour at room temperature. The membranes were washed with PBS-T and incubated with streptavidin-conjugated horseradish peroxidase (Jackson ImmunoResearch, West Grove, Pennsylvania, USA) at 1:500 in 1% NFDM in PBS-T for 1 hour at room temperature. After washing, immunoreactivity was detected using enhanced chemiluminescence solutions (Bio-Rad, USA) and the Bio-Rad ChemiDoc MP imaging system. Western blots to assess quality of EVs isolated with SEC were performed in 4 biological replicates per sex.

### Transmission Electron Microscopy

Total of 5 µL of sample were adsorbed onto glow-discharged 200-mesh carbon-coated Cu grids for 3 minutes. The excess was wicked off with Whatman filter paper. The grids were washed 3 times with MilliQ water and then negatively stained with 2% uranyl acetate for 45 seconds. The excess was wicked off with Whatman filter paper. Images were then acquired on a FEI Tecnai G2 Spirit Twin 120 kV Cryo-TEM. For immunolabeling, 5 µL of sample was adsorbed onto glow-discharged 200-mesh carbon-coated Cu grids for 5 minutes. The excess was wicked off with Whatman filter paper, and the grid was situated (sample-side down) onto a droplet of 4% paraformaldehyde for 10 minutes. It was then washed on 2 droplets of PBS, 5 minutes each. It was incubated on 1.1% NH_4_Cl for 10 minutes and washed again on 2 droplets of PBS, 5 minutes each. The grid was blocked with 1% NFDM for 20 minutes and then incubated with anti-CD81 (System Biosciences, USA, Cat#EXOAB-KIT-1) (1:20) for 30 minutes at room temperature. It was washed on 3 droplets of PBS, 5 minutes each. After that, it was incubated with gold-conjugated anti-rabbit antibody (Abcam, UK, Cat#ab27234) (1:20) at room temperature for 1 hour. It was washed on 3 droplets of PBS, 5 minutes each and fixed with 4 % paraformaldehyde for 5 minutes. Three consecutive washes were performed, each for 5 minutes, with 1 droplet of PBS followed by 2 droplets of MilliQ water. The sample was negatively stained with 2% uranyl acetate for 30 seconds and was finally left to air dry. Images were then acquired on a FEI Tecnai G2 Spirit BioTwin 120 kV Cryo-TEM. As control experiments, 1 grid was prepared similarly but was incubated with anti-IgG (1:20) instead of anti-CD81, while another grid was prepared similarly but without incubation with primary antibody. The electron microscopy was performed at the McGill University Facility for Electron Microscopy Research.

### RNA Extraction

RNA was extracted from a 50-µL aliquot of F1 (EV fraction) from each subject. Briefly, samples were incubated with 25 µg of Proteinase K (New England Biolabs, Ipswich, Massachusetts, USA) at 37°C for 30 minutes. They were then incubated with 50 µg of PureLink RNAseA (ThermoFisher Scientific, USA) to eliminate ambient RNA, at room temperature for 2 minutes. QIAzol lysis reagent was added at 5X to each sample, which were then incubated at room temperature for 5 minutes. Then, 1X chloroform was added, and the samples were incubated at room temperature for 3 minutes. They were centrifuged at 12 000 *g* at 4°C for 15 minutes, and the aqueous phase was collected. After that 1.5 volumes of 100% ethanol were added and mixed gently. The mixture was then added to QIAGEN MinElute columns, and the MiRNeasy Micro Kit was used according to the manufacturer’s protocol (QIAGEN, Germany). RNA was quantified using a Quant-iT RiboGreen RNA Assay (ThermoFisher Scientific, USA).

### Library Preparation and Sequencing

Small RNA libraries were constructed with equivalent amounts of RNA from F1 (EV fraction) of each subject using the Galas Lab–4N library prep–Version 1.0 protocol (Giraldez et al., 2018). Briefly, a 3’ degenerate adaptor was ligated to the RNA, followed by the ligation of a 5’ degenerate adaptor. cDNA was then synthesized and amplified for 10 cycles with Illumina indices. The quality and quantity of cDNA was assessed using an Agilent TapeStation System with high-sensitivity DNA chips (Agilent Technologies, Santa Clara, California, USA). Equivalent amounts of cDNA from each subject were pooled together. Adaptor and primer dimers were removed using BluePippin gel purification (Sage Science, Beverly, Massachusetts, USA), and the cDNA was amplified for 10 more cycles. Adaptor and primer dimers were removed again using BluePippin gel purification. Libraries were sequenced at the McGill University and Genome Quebec Innovation Centre on Illumina HiSeq 4000 (male cohort) and on Illumina NOVASeq 6000 (female cohort).

### RNA Biotype Identification

The counts for each biotype were produced by excerpt and converted to counts per million. Plots were generated using ggplot2 v3.4.0 (Wickham, 2016).

### miRNA Differential Expression Analysis

Reads were trimmed using cutadapt (male cohort) and bbduk.sh from bbmap v38.86 (female cohort) to remove technical sequences. Trimmed reads were input into the Extracellular RNA Communication Consortium’s exceRpt small RNA-Seq pipeline (v.4.6.3), which aligned the reads to the human genome and quantified the various miRNAs into counts. Only reads that matched the reference genome perfectly were considered aligned. Only miRNAs with more than 10 counts in more than 70% of the subjects in either the CTRL or MDD group were retained for further analysis. The sequencing batch effect was assessed using principal component analysis, and the variance between the cohorts was high, so this effect could not be differentiated from sex effect and the sexes were analyzed separately. Normalization and differential expression analysis were performed using DESeq2 v1.34.0, which implements the median of ratios normalization method and a negative binomial general linear model to model the effect of factors of interest on expression. Covariates were chosen for inclusion in DESeq2’s general linear model based on associations between the top 5 principal components and covariates, violin plots produced by variancePartition v1.24.0 of the variance explained by each covariate for each miRNA (supplementary Fig. 1), and histograms of *P* values for the association of covariates with miRNAs. Factors that significantly differed between CTRL and MDD (PMI in males and age in females) were not correlated to the expression levels of the miRNAs of interest (supplementary Fig. 2). For the male cohort, lane and isolation batch were used as covariates, while for the female cohort, pH and isolation batch were used as covariates. *P* values adjusted for multiple testing were obtained from R’s p.adjust function with method = “BH” (Benjamini-Hochberg). The threshold for statistical significance was set at *P* ≤ .05. R version 4.1.2 was used for all analyses.

### Real-Time PCR Quantification

RNA isolated from EVs was used to synthesize cDNA using the TaqMan Advanced miRNA cDNA Synthesis Kit (ThermoFisher Scientific, USA) according to the manufacturer’s protocol, except for the miR-Amp reaction step where 18 cycles were used. Real-time PCR (qPCR) was run in triplicate using the QuantStudio 6 Flex System, and the data were collected using the QuantStudio Real-Time PCR Software v1.7.1 (Applied Biosystems, Waltham, Massachusetts, USA). To measure miRNA expression, we used TaqMan Advanced miRNA Assays (supplementary Table 1) with TaqMan Fast Advanced Master Mix (ThermoFisher Scientific, USA). Expression levels were calculated using the absolute (standard curve) quantification method. TaqMan Advanced miRNA Assays (ThermoFisher Scientific, USA) for hsa-miR-9-5p, hsa-miR-103-3p, hsa-miR-92a-3p, hsa-miR-129-5p, hsa-miR-499a-5p, hsa-let-7e-5p, and hsa-miR-132-5p. Hsa-miR-9-5p and hsa-miR-103a-3p were used as endogenous controls based on lowest variability among subjects, determined by NormFinder software (Andersen et al., 2004).

### Statistical Analysis

#### Correlation Analyses

Correlation analyses were performed using GraphPad Prism 8. For correlations between qPCR and sequencing results, qPCR expression values were determined from the standard curve quantification method and were normalized to the geometric mean expression of the endogenous controls hsa-miR-9-5p and hsa-miR-103a-3p. These values were correlated with normalized sequencing counts in the same subjects. For correlations between EVs and bulk sequencing, normalized sequencing counts for EVs were correlated with normalized counts from the RNA sequencing dataset from bulk ACC of some overlapping subjects from (Fiori et al., 2021). Correlations were done using Pearson or Spearman correlation tests where appropriate. Shapiro-Wilk test was used to assess normality.

#### Student’s *t* Test

One-tailed Student *t* test was used to compare the normalized expression levels between CTRL and MDD of hsa-miR-129-5p in males and hsa-miR-92a-3p in females. GraphPad Prism 8 was used for analysis. Shapiro-Wilk test was used to assess normality.

#### Target Prediction and Functional Analysis

Target mRNAs of the top miRNAs for males and females were predicted using 5 databases: miRWalk (version 3.0), mirDIP (version 5.2.8.1), miRDB, RNA22 (versions 1.0 and 2.0), and DIANA-microT-CDS. Genes common between all 5 databases were considered as targets. Gene ontology enrichment analysis for biological processes was performed using clusterProfiler R. Protein-protein interaction network analysis for the predicted targets was performed using STRING (version 11.5). Clusters were determined using the kmeans clustering (5 clusters) option.

## RESULTS

### EV Isolation and Quality Assessment

**Figure 2.**
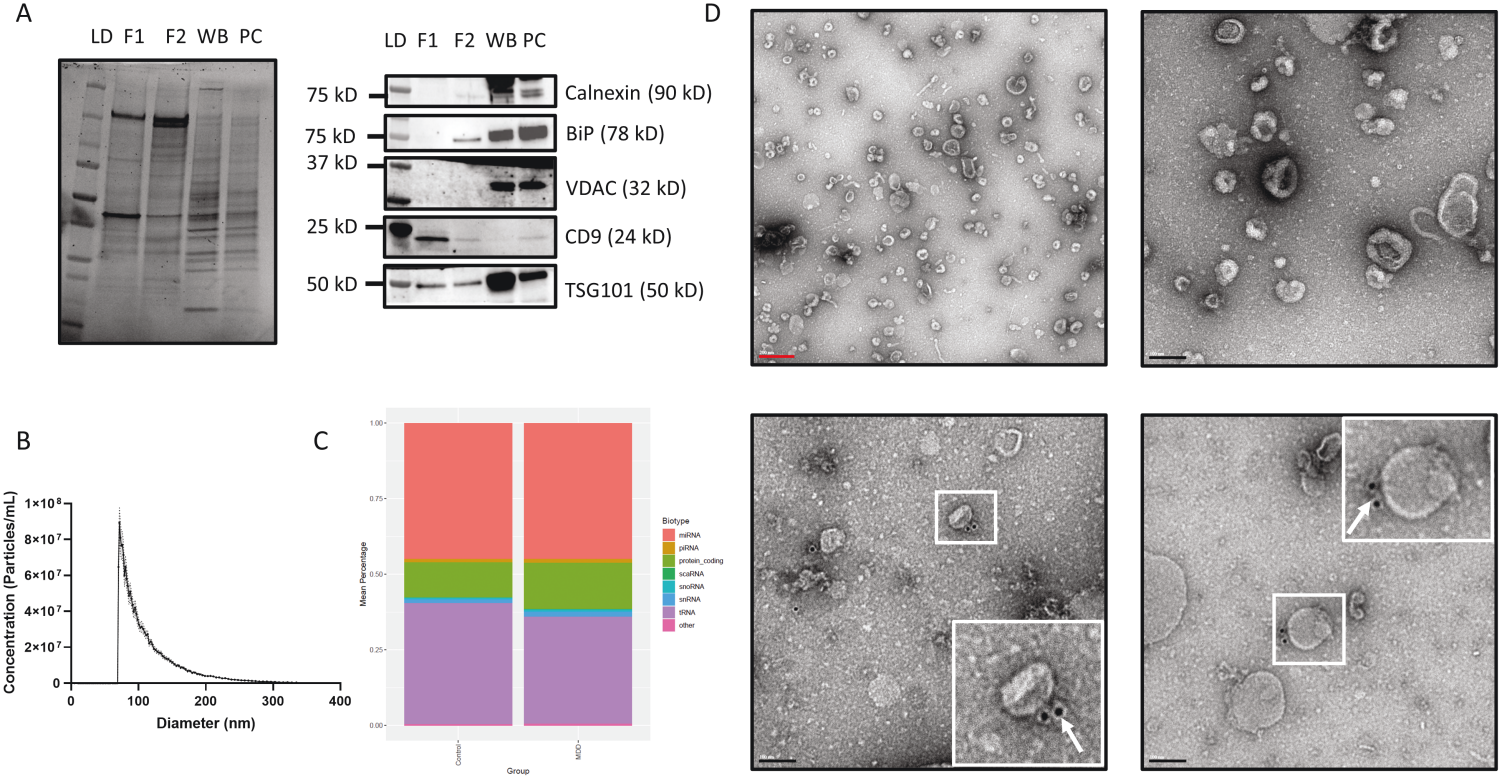
Quality assessment of EV isolation. (A) Left: Representative stain-free image showing total protein loading for western blot. Right: Representative western blot for Calnexin, BiP, VDAC, CD9, and TSG101 in F1, F2, WB, and PC. (B) Average distribution of particle size from 20 subjects (5 M-CTRL, 5 M-MDD, 5 F-CTRL, 5 F-MDD). (C) Graph showing RNA biotype precent distribution in CTRL and MDD groups. (D) Top: Negative stain TEM images of F1. Bottom: TEM images of EVs immunolabelled with anti-CD81. White rectangles are a zoomed in image of labelled EVs, while white arrows indicate electron-dense gold particles, which are seen as black dots, labeling CD81 on the surface of EVs. Scale: red bar = 200 nm; black bar = 100 nm. F1, fraction 1 (EV fraction); F2, fraction 2 (protein fraction); LD, ladder; PC, pellet with collagenase; WB, whole brain homogenate.

EVs were isolated following the protocol shown in Figure 1 and were assessed using western blots. The blots showed absence of contaminating proteins from the endoplasmic reticulum (ER), Calnexin and Binding immunoglobulin protein (BiP), and from the mitochondria, Voltage dependent anion channel (VDAC) (Figure 2A, right; supplementary Fig. 3). Specifically, the F1 fraction, where we expected our vesicles of interest to be, showed no contamination compared with the WB and PC. On the other hand, Tumor susceptibility gene 101 (TSG101) and CD9, which are known markers of EVs, were present in F1. CD9 was enriched in F1 compared with all other fractions, indicating a successful enrichment of EVs from postmortem tissue. Because it is difficult to select an endogenous control that is uniformly expressed among the different samples used, total protein loading was used to control for equal input in western blots (Figure 2A, left).

We characterized the size distribution of our vesicles using microfluidic resistive pulse sensing. Figure 2B shows that the main distribution of particles in F1 is less than 200 nm in diameter with only a small tail greater than 200 nm, consistent with small EVs. This size distribution, ranging in size between 30 and 200 nm in diameter, was confirmed using TEM, which also indicated that our EVs had the typical morphology with minor contamination (Figure 2D, top). We performed immunolabeling of our EVs using an antibody against CD81, a transmembrane protein found on the surface of EVs (Figure 2D, bottom), controlling for the specificity of both the primary and secondary antibodies (supplementary Fig. 4).

Additionally, our test of EV isolation using UC showed that small EVs were found predominantly in UC-F2 but with major contamination, as indicated by the presence of Calnexin, BiP, VDAC, TSG101, and CD9 in western blots as well as in TEM images (supplementary Fig. 5). UC-F3 contained small vesicles, with minor contamination, but they did not carry CD9 or TSG101, whereas UC-F1 contained much larger vesicles (supplementary Fig. 5).

### Small RNA Profiling and Group Comparisons

In an attempt to acquire a broad portrait of RNA cargo profiles of brain EVs in depression, we first looked at the proportions of RNA biotypes carried in EVs in each group. Consistent with previous reports (Sadik et al., 2018), we found miRNA and tRNA were the most abundant RNA biotypes in EVs, followed by protein coding RNAs, regardless of group. Other biotypes, such as piwi-interacting RNA, small nucleolar RNA, and small nuclear RNA, were also present but at much lower proportions (Figure 2C).

**Figure 3.**
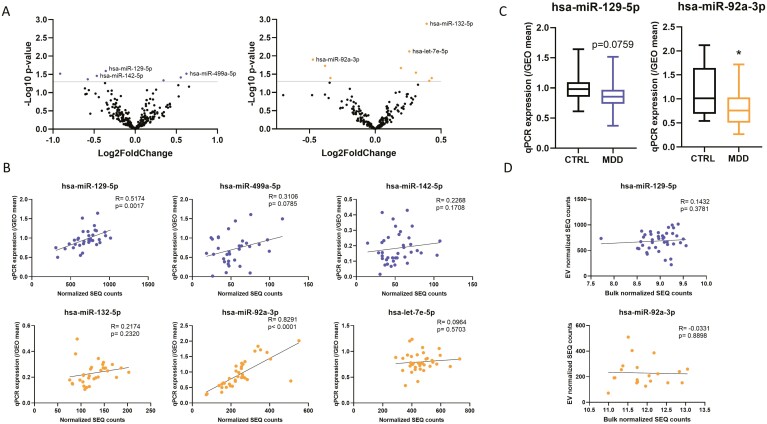
Comparative analysis of EV miRNAs in males and females. (A) Volcano plots showing altered miRNA expression for males (blue) and females (orange) at a cut-off of *P* value ≤ .05. (B) Correlation plots between normalized sequencing counts and qPCR expression of the 3 top miRNAs for males (blue) and females (orange). (C) Boxplots showing differences in normalized qPCR expression levels between CTRL and MDD for miR-129-5p in males and miR-92a-3p in females. (D) Correlation plots between normalized sequencing counts from bulk tissue from Fiori et al. (2021) and normalized sequencing counts from overlapping subjects. Results for miR-129-5p (blue) and miR-92a-3p (orange) are shown. CTRL, control; GEO, geometric; MDD, major depressive disorder; SEQ, sequencing.

Given that miRNAs are the most studied biotype in EVs and that they have been previously implicated in the etiopathogenesis of MDD (Żurawek and Turecki, 2021), we opted to focus on miRNA. Our initial exploratory analysis indicated potential differences in miRNA levels in both sexes (Table 2; Figure 3A). We selected the top 3 most significant hits for each sex for follow-up by qPCR. Due to the lack of a reliable Taqman advanced miRNA assay for miR-320d, we opted to measure the next top hit, miR-142-5p, in males. The expression levels of 2 miRNAs, miR-129-5p and miR-92a-3p, were validated. Specifically, we found that miR-129-5p qPCR expression correlated (*P *= .0017; R = 0.5174) with that of sequencing in males (Figure 3B, top), and in females, miR-92a-3p qPCR expression correlated (*P* < .0001; R = 0.8291) with that of sequencing (Figure 3B, bottom). Furthermore, comparisons between groups showed concordant decreased expression of miR-92a-3p in females but not in males and of miR-129-5p (trending) in males but not in females (Figure 3C, females: miR-92a-3p, *P* = .0152, miR-129-5p, *P* = .2497; males: miR-129-5p, *P* = .0759, miR-92a-3p, *P* = .5829, supplementary Fig. 6).

**Table 2.** Top altered brain EV miRNAs in males and females

	Upregulated				Downregulated			
	miRNA	log2 Fold Change	*P* value	Q value	miRNA	log2 fold change	*P* value	Q value
Females	miR-132-5p	0.39	.0013	0.2390	miR-92a-3p	−0.47	.0127	0.7676
	let-7e-5p	0.26	.0075	0.6840	miR-885-5p	−0.38	.0186	0.7809
	let-7f-5p	0.20	.0215	0.7809	miR-708-5p	−0.34	.0401	0.9211
	miR-126-5p	0.31	.0288	0.8747				
	miR-320b	0.43	.0405	0.9211				
	miR-421	0.41	.0479	0.9454				
							
Males	miR-499a-5p	0.62	.0302	0.9896	miR-129-5p	−0.36	.0255	0.9896
	miR-369-5p	0.55	.0382	0.9896	miR-320d	−0.91	.0303	0.9896
	miR-195-5p	0.34	.0462	0.9896	miR-142-5p	−0.47	.0347	0.9896
					miR-3065-5p	−0.58	.0430	0.9896

miRNAs showing nominally significant expression in males or females are shown.

To determine whether these effects are specific to EVs, we correlated the expression of miRNAs from EVs to miRNA expression levels observed in bulk ACC tissue in the subjects that overlap with those from a previous study of our group (Fiori et al., 2021). Figure 3D and supplementary Fig. 7 show that there was no correlation between expression of these miRNAs in EVs and in bulk tissue (females miR-92a-3p, *P* = .8898 and R = −0.0331; males miR-129-5p, *P* = .3781 and R = 0.1432).

Finally, to identify the potential functional roles of miR-129-5p and miR-92a-3p, we performed an in silico analysis to predict and characterize their target mRNAs (supplementary Table 2). Each list of potential mRNA targets was analyzed using STRING (supplementary Fig. 8A and C; supplementary Table 3) and clusterProfiler R (supplementary Fig. 8B and D; supplementary Table 4) to identify their biological roles. Altogether, our analyses suggest that miR-92a-3p regulates genes related to neurotransmission, synapses, and ion transport, while miR-129-5p regulates genes related to axon guidance and Bone Morphogenic Protein signaling, indicating an involvement in synaptic plasticity (Meyers and Kessler, 2017).

## DISCUSSION

In this study, we isolated EVs from postmortem brain tissue of individuals who had depression and died by suicide as well as matched controls who died suddenly and who had no psychiatric illness. Our study is the first, to our knowledge, to employ SEC to isolate EVs from postmortem brain tissue on a large scale. Conventionally, UC is the most commonly used technique to isolate EVs (Gardiner et al., 2016); however, it has several disadvantages, such as the presence of soluble protein contamination as well as EV aggregation at high centrifugal forces (Linares et al., 2015; Crescitelli et al., 2021). Another disadvantage is the complexity and low reproducibility of the procedure (see review Konoshenko et al., 2018), which is not favorable for studies with larger cohorts. Indeed, we attempted to employ UC for EV isolation using the protocol developed by Vella et al. (2017), in 2 technical replicates. Along with the technical challenges, we were unable to reproduce EVs of similar quality to what was expected (supplementary Fig. 5). In performing our adapted SEC-based protocol, we found it yielded more consistent results (supplementary Fig. 3) allowing for more streamlined EV isolation, which is crucial for large sample sizes like the one used in the current study. We enriched small EVs that were not the result of self-assembled membrane fragments from membranous organelles, such as the ER and mitochondria. Collectively, our results indicate that our adapted method can successfully isolate high-quality EVs from postmortem human brain tissue and allows a more streamlined approach to studying EVs in similar experimental designs.

We found that the biotype distribution was consistent with previous reports (Sadik et al., 2018). In our sequencing results, mRNAs were detected, and this is not surprising given that mRNAs have been reported in EVs both in full length (Valadi et al., 2007; Morhayim et al., 2017) and in fragments (Batagov and Kurochkin, 2013; Perez-Boza et al., 2018). We expect that mRNA fragments are more likely to be present in our EV samples, as we performed small-RNA sequencing. Biotypes that are much larger than miRNA, such as tRNA and small nucleolar RNA, were also detected, and this is common with small-RNA library preparation kits, which are known to detect mRNAs as well (Yeri et al., 2018; Srinivasan et al., 2019). Additionally, the use of degenerate adaptors during our library preparation removes the bias toward certain RNA sequences, allowing different biotypes to be detected (Giraldez et al., 2018). On the other hand, other RNA biotypes known to be present in EVs, such as circular RNA and long noncoding RNA (Lai et al., 2022), were not detected in our data. In our study, we focused on the most commonly studied and abundant EV RNA cargo: miRNA. Future studies should evaluate whether other RNA biotypes are dysregulated in EVs in MDD, as they have also been shown to be implicated in depression and other psychiatric illnesses (Huang et al., 2017; Li et al., 2020; Yoshino and Dwivedi, 2020; Lin et al., 2021).

Our study is one of the first to examine not only potential alterations of miRNAs in both males and females but also brain EVs in the context of depression. Although little is known about sex-specific factors relating to the role of miRNAs in depression (Hodes et al., 2017), sex differences in miRNA regulation in other conditions, such as stress (Krispil-Alon et al., 2022), Tau pathology (Kodama et al., 2020), ASD (Schumann et al., 2017), and schizophrenia (Ragan et al., 2017), have been documented in the brain. Sex-specific transcriptomic dysregulation in the depressed brain has also been reported (Labonte et al., 2017). Our initial analysis identified differences in miR-129-5p in depressed males and in miR-92a-3p in depressed females. While there is no literature regarding sex-specific roles of either miRNA, interestingly, miR-92a-3p is coded on both chromosomes 13 and X, which might be related to its alteration in females specifically. In fact, according to our data, females generally had higher levels of miR-92a-3p in brain EVs compared with males, so one could speculate that a decrease in this miRNA might have greater consequences in females. Although we found trending alterations in the levels of different miRNA species in males (miR-129-5p) and females (miR-92a-3p), we cannot be sure whether this difference is explained solely by sex or also by technical differences, as each sex was sequenced on a different platform. Future studies are warranted to confirm these sex differences.

Furthermore, we found that the EV levels of these miRNAs did not correlate with bulk tissue levels (Figure 3C). This could suggest specific packaging of cargo into EVs, as they do not directly mirror the bulk tissue they came from, carry a unique repertoire of miRNAs, and could provide a different level of information than bulk tissue. Indeed, there is ample evidence supporting cargo sorting into EVs (see review by Chen et al., 2021b).

Finally, we performed functional follow-up to explore the possible biological functions of miR-92a-3p and miR-129-5p. We found that both miRNAs regulate genes related to synaptic plasticity and neurotransmission. In fact, each of these miRNAs has been previously implicated in brain function and disorders, including depression. MiR-92a-3p has been associated with poststroke depression (Zhang et al., 2016; He et al., 2017), Alzheimer disease (Siedlecki-Wullich et al., 2019; Pena-Bautista et al., 2022), Parkinson disease (Taguchi and Wang, 2018), ASD (Trivedi et al., 2022), and schizophrenia (Ma et al., 2018). Chen et al. (2021a) reported that the expression levels of miR-92a-3p, alongside miR-129-5p, in plasma EVs were negatively correlated with Hamilton-Depression and Hamilton-Anxiety rating scores in patients with substance use disorders. MiR-92a-3p has also been implicated in animal models of stress and depressive-like behaviors (Jin et al., 2016; Munoz-Llanos et al., 2018; Ji and Zhao, 2023). Moreover, miR-92a-3p regulates genes that are important for synaptic function (Huang et al., 2006; Morgan et al., 2010; Blair and Harvey, 2012; Gross and Bassell, 2014; Letellier et al., 2014; Li et al., 2017; He et al., 2020; Bressler et al., 2021; Trivedi et al., 2022; Ji and Zhao, 2023). Collectively, the literature points toward a clear involvement of miR-92a-3p in brain disorders and, more specifically, synaptic function.

miR-129-5p has been similarly implicated in several brain disorders, including Alzheimer’s (Dobricic et al., 2022; Gupta and Kumar, 2022), Parkinson's (Dobricic et al., 2022), epilepsy (Sosanya et al., 2015; Liu et al., 2017; Wan and Yang, 2020), and ALS (Hawley et al., 2020; Loffreda et al., 2020). In a study by Videtiĉ Paska et al. (2022), miRNAs that target genes associated with suicidality were predicted, and miR-129-5p was one of them, although no difference was found in its expression levels between suicide completers and control subjects. It is interesting that our exploratory findings suggest a possible reduction in miR-129-5p in brain EVs, specifically, in suicide completers. Additionally, miR-129-5p was found to be altered in several brain structures of animals in different stress paradigms and other models (Buran et al., 2017; Chang et al., 2021; Qinlin et al., 2022a, 2022b; Yasir et al., 2022). Similar to miR-92a-3p, we identified that miR-129-5p is important for neuronal function. It is expressed at high levels in synaptosomal preparations, and it regulates genes that play crucial roles at the synapse (Zongaro et al., 2013; Buran et al., 2017; Rajman et al., 2017; Wu et al., 2019; Glaesel et al., 2020). miR-129-5p is also involved in neurogenesis and axon guidance (Trujillo-Gonzalez, et al., 2019; Wu et al., 2019; Loffreda et al., 2020; Wan and Yang, 2020). Moreover, another important mechanism that has been linked to depression and is affected by miR-129-5p is neuronal autophagy (Qinlin et al., 2022a, 2022b). Finally, miR-129-5p regulates neuronal function through the regulation of voltage-gated potassium ion channel Kv1.1 (Sosanya et al., 2015). Collectively, these results suggest that these miRNAs seem to be involved in similar functions related to neurotransmission and synaptic plasticity, both of which could be impaired in depression. According to the literature, abnormal levels of these miRNAs, whether too high or too low, might be related to disease development. In addition, both miRNAs have been implicated in inflammation, a process that is also thought to be important in the pathophysiology of depression (Beurel et al., 2020; Chang et al., 2021; Fujiwara et al., 2022; Zhou et al., 2022).

In summary, we used an unbiased, streamlined approach to profiling miRNAs from EVs isolated from brain tissue. qPCR follow-up on the top hits suggested that these miRNAs do indeed show a decrease in the context of depression; however, future studies with a larger sample size are required to confirm whether miR-129-5p and miR-92a-3p are dysregulated in depressed males and females, respectively.

## Supplementary Material

pyae013_suppl_Supplementary_Figures_S1-S8

pyae013_suppl_Supplementary_Tables_S1

pyae013_suppl_Supplementary_Tables_S2

pyae013_suppl_Supplementary_Tables_S3

pyae013_suppl_Supplementary_Tables_S4

## Data Availability

The data underlying this article are available in GEO at https://www.ncbi.nlm.nih.gov/geo/ and can be accessed with accession numbers GSE255478 and GSE255480.
