# Supplementary material for: Profiling Small RNA From Brain Extracellular Vesicles in Individuals With Depression
Source: Int J Neuropsychopharmacol. 2024 Mar 8;27(3):pyae013. doi: 10.1093/ijnp/pyae013 (PMC10946232; doi:10.1093/ijnp/pyae013)
Supplement: pyae013_suppl_Supplementary_Figures_S1-S8 [file pyae013_suppl_supplementary_figures_s1-s8.docx]

**Supplementary Figures**


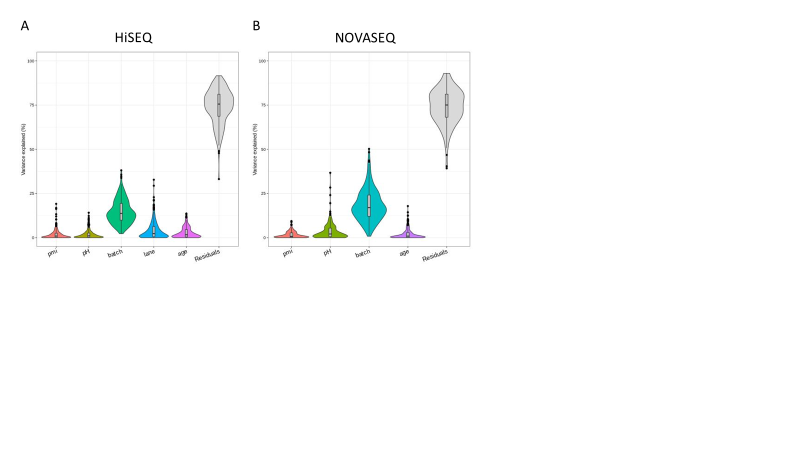


**Supplementary Figure S1.** Violin plots of the percentage of variation explained by each covariate for each miRNA for (A) HiSEQ and (B) NOVASEQ.


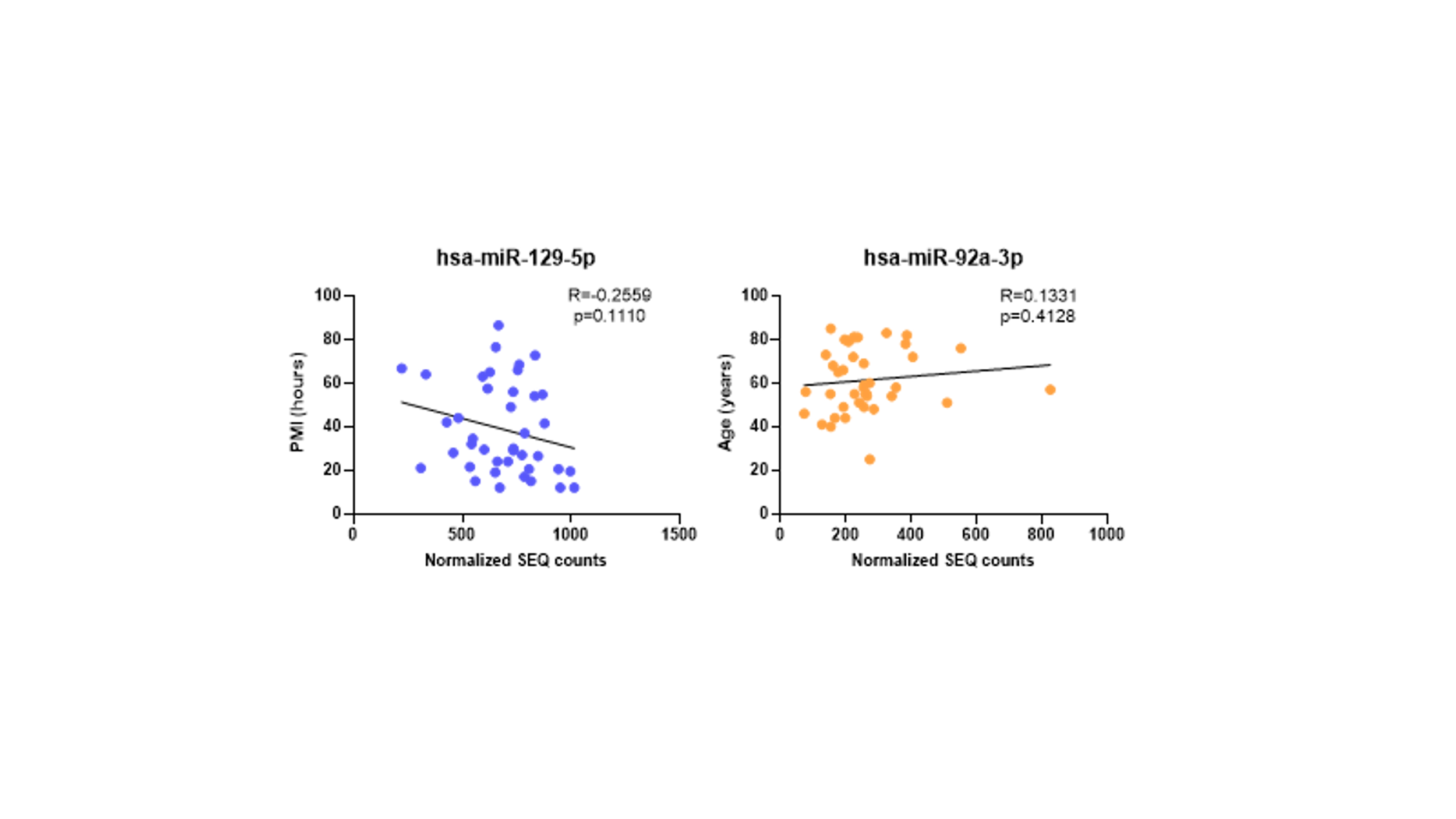


**Supplementary Figure S2.** Correlation plots between PMI and normalized sequencing counts of miR-129-5p in male EVs and between age and normalized sequencing counts of miR-92a-3p in female EVs.


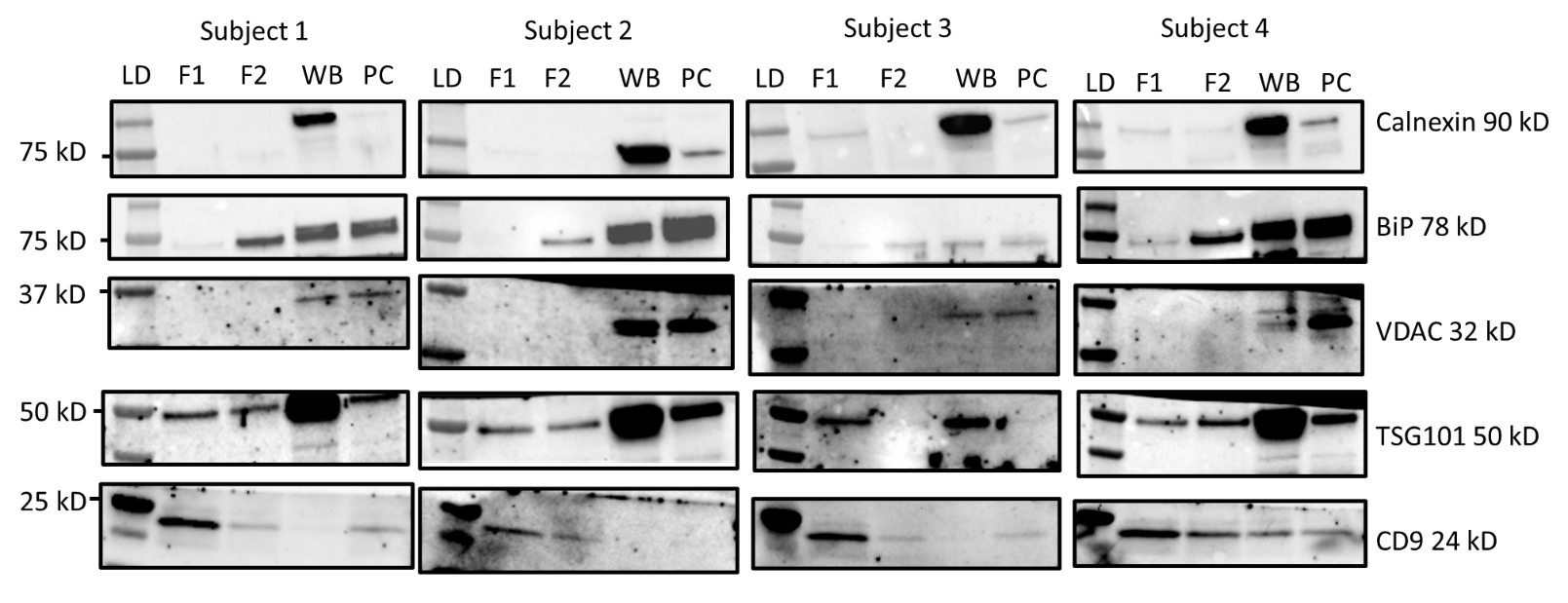


**Supplementary Figure S3.** Western blots of four biological replicates for Calnexin, BiP, VDAC, TSG101, and CD9. LD: ladder; WB: Whole brain homogenate; PC: Pellet with collagenase from first spin; F1: Fraction 1 (EV Fraction); F2: Fraction 2 (Protein Fraction); kD: KiloDalton.


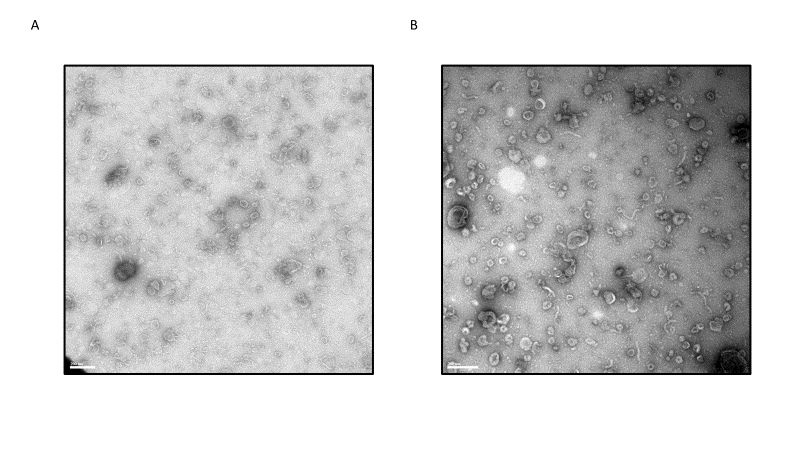


**Supplementary Figure S4.** TEM images for negative control experiments using (A) no primary antibody and (B) anti-IgG antibody to show the specificity of the primary and secondary antibodies used in our experiment. White bar: 200 nm scale.


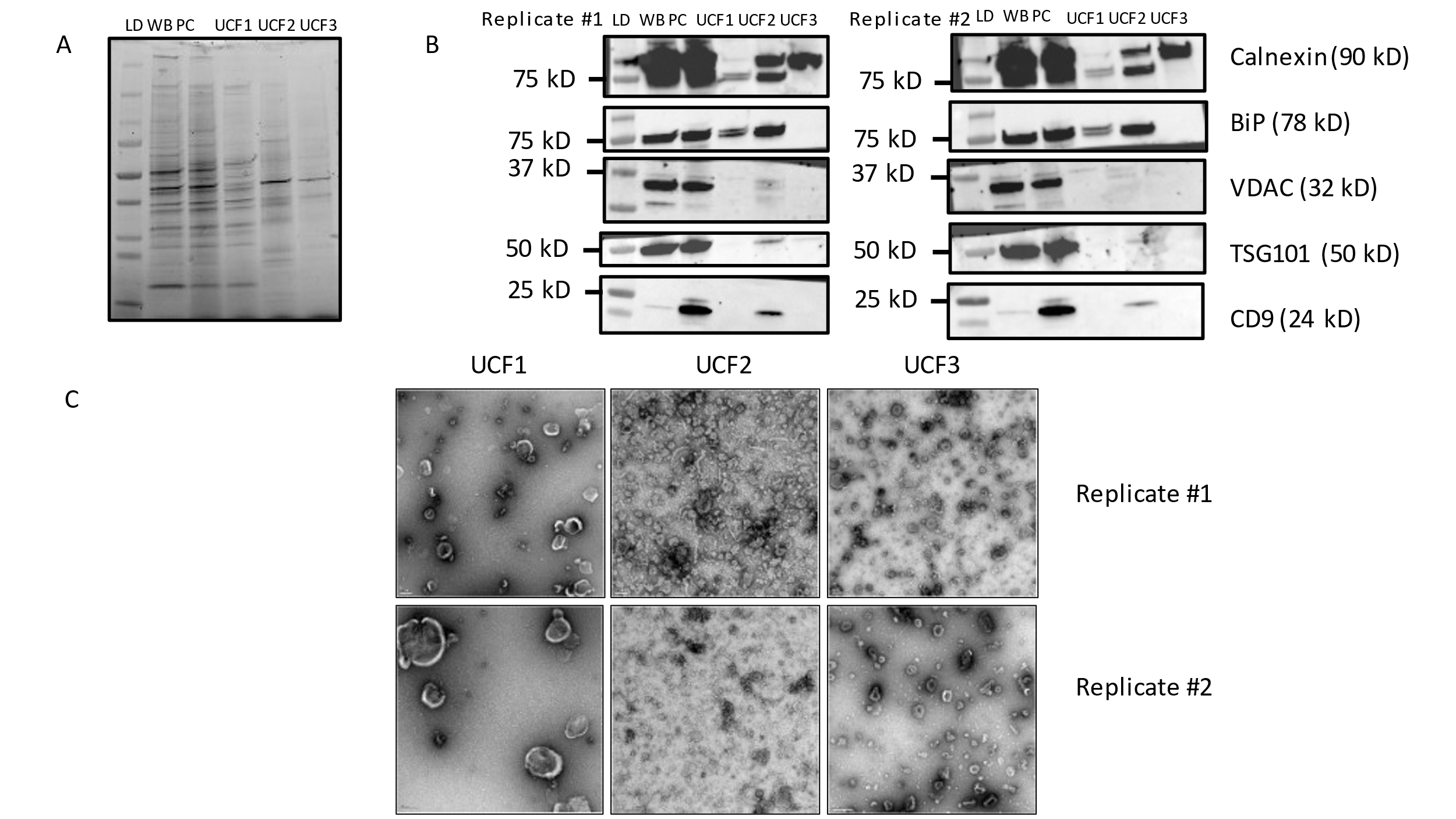


**Supplementary Figure S5.** Quality assessment of EV extraction using the protocol by Vella et al., 2017, unmodified (ultracentrifugation). (A) Representative stain-free image showing total protein loading for western blot. (B) Western blot in two biological replicates for Calnexin, BiP, VDAC, CD9, and TSG101 in UCF1, UCF2, UCF3, WB, and PC. (B) Negative stain TEM images of UCF1, UCF2, and UCF3. White bars: scale; for UCF1and UCF3: 200 nm; for UCF2: 100 nm scale. LD: Ladder; UCF1: ultracentrifugation fraction 1; UCF2: ultracentrifugation fraction 2; UCF3: ultracentrifugation fraction 3; WB: Whole brain homogenate; PC: Pellet with collagenase; kD: KiloDalton.


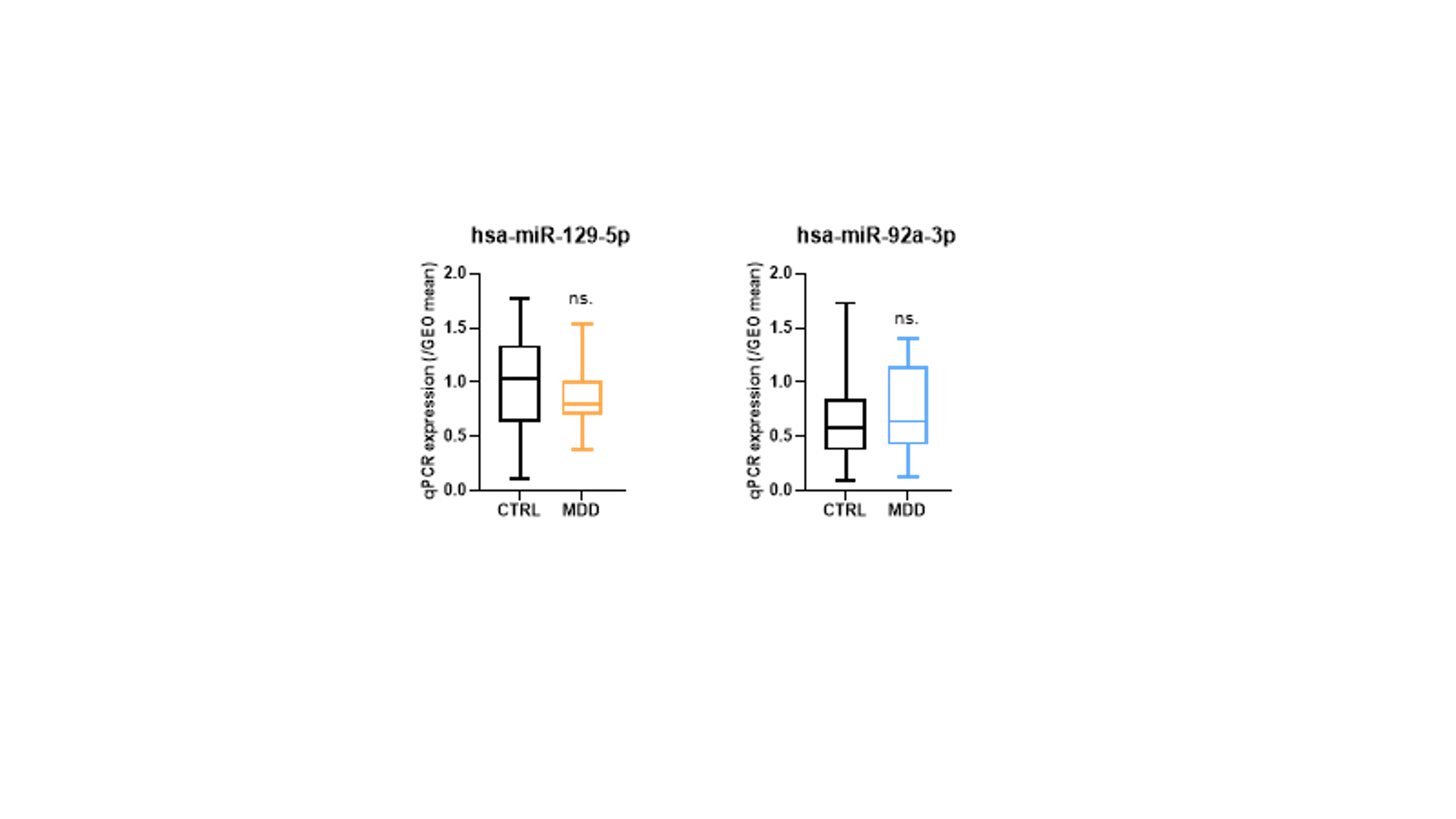


**Supplementary Figure S6.** Boxplots showing differences in normalized qPCR expression levels between CTRL and MDD EVs for miR-129-5p in females (p=0.2497) and miR-92a-3p in males (p=0.5829).


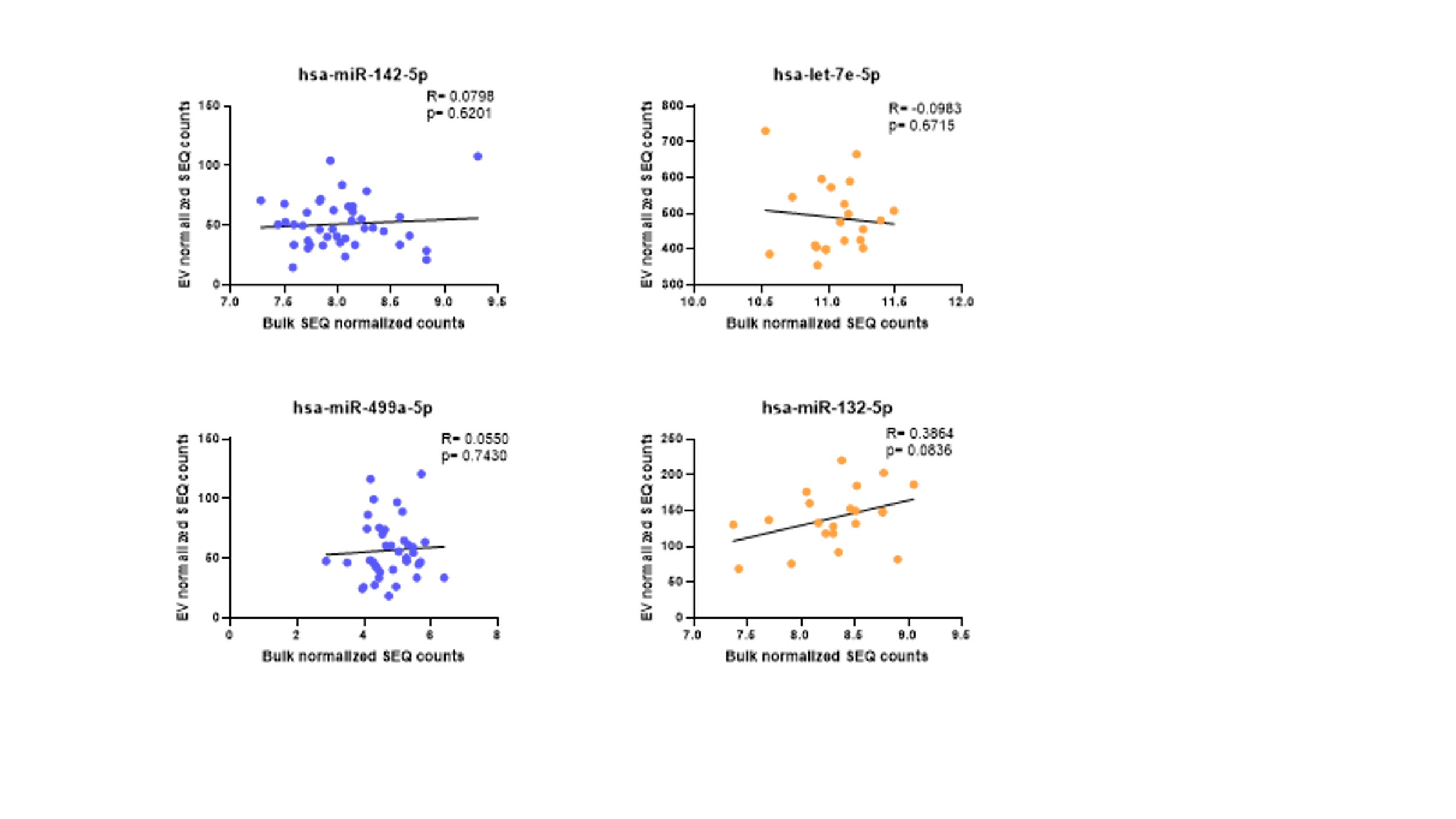


**Supplementary Figure S7.** Correlation plots between normalized sequencing counts from bulk tissue from Fiori et al., (2020) and normalized sequencing counts from our study in a subsample of overlapping subjects. Results for miR-142-5p and miR-499a-5p (male hits, blue) and let-7e-5p and miR-132-5p (female hits, orange) are shown. SEQ: sequencing; GEO: geometric.


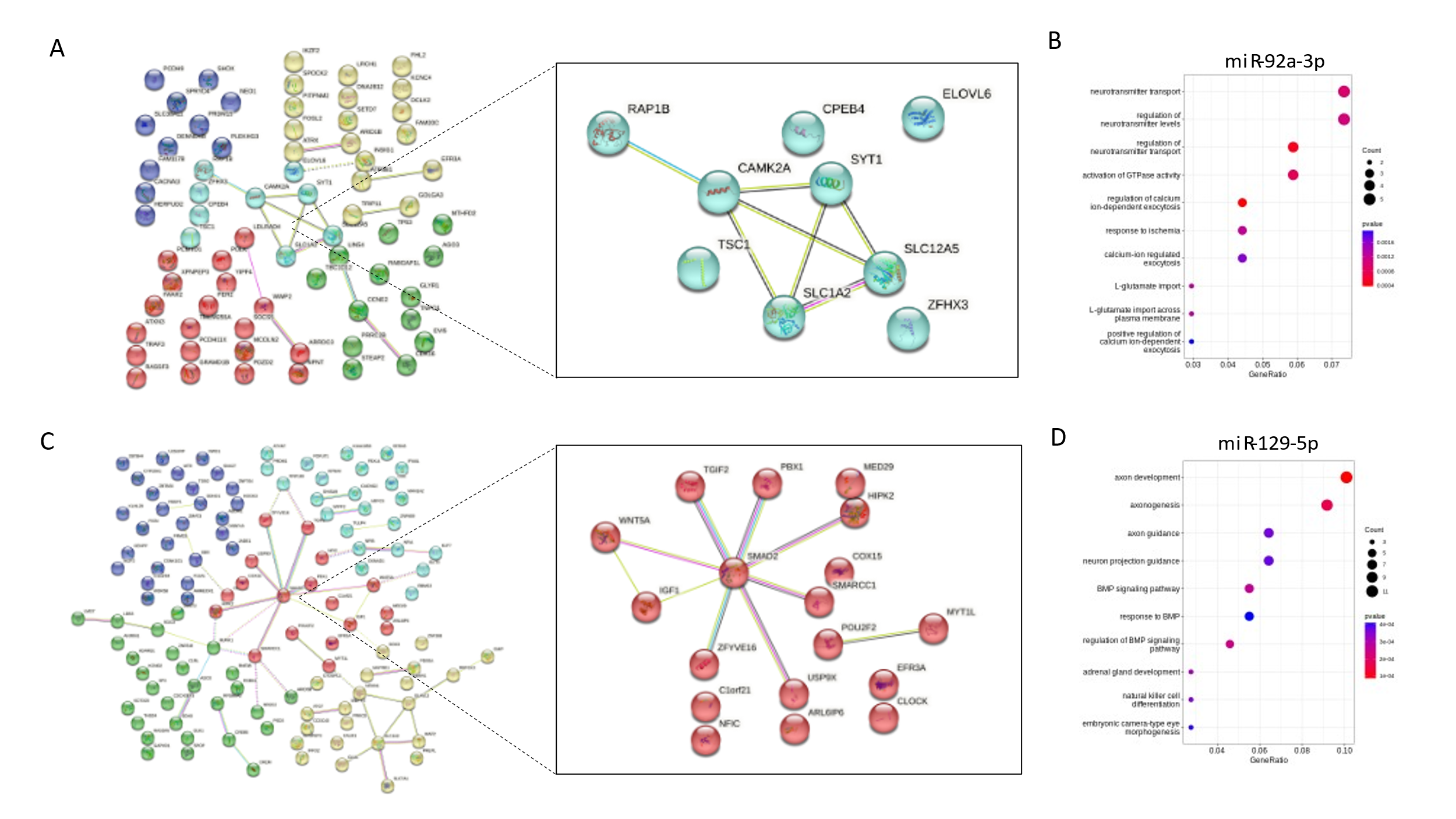


**Supplementary Figure S8.** STRING networks of mRNA targets of (A) miR-92a-3p and (C) miR-129-5p. The most connected networks are highlighted. GO enrichment analysis of mRNA targets of (B) miR-92a-3p and (D) miR-129-5p.
